# Analysis of the occurrence of deep venous thrombosis in lower extremity fractures: A clinical study

**DOI:** 10.12669/pjms.344.14752

**Published:** 2018

**Authors:** Qiang Li, Xiao Chen, Yuanyuan Wang, Lin Li

**Affiliations:** 1 Qiang Li, Vascular Surgery Department, Qingdao Hiser Medical Group, Qingdao, Shandong Province, 266033, China; 2 Xiao Chen, Vascular Surgery Department, Qingdao Hiser Medical Group, Qingdao, Shandong Province, 266033, China; 3 Yuanyuan Wang, Vascular Surgery Department, Qingdao Hiser Medical Group, Qingdao, Shandong Province, 266033, China; 4 Lin Li, Vascular Surgery Department, Qingdao Hiser Medical Group, Qingdao, Shandong Province, 266033, China

**Keywords:** Deep venous thrombosis (DVT), Fracture site, Lower extremity, Risk factors

## Abstract

**Objective:**

To clarify if fracture site is correlated to the occurrence of deep venous thrombosis, and determine the risk factors of deep venous thrombosis in lower extremity fractures, help surgeons make prophylaxis for the disease correctly.

**Methods:**

The patients with lower extremity fractures treated surgically in the orthopedics department of our hospital from May 2012 to July 2017 were reviewed retrospectively. The clinical data including age, gender, fracture site, surgery modality, hospital stay, operation time, occupation type, hypertension, coronary heart disease, diabetes, smoking status, drinking status, postoperative exercises were collected and analyzed.

**Results:**

Eight hundred and twenty-nine patients were included for analysis, in which 68 were included in deep venous thrombosis group, 761 were included in the non-deep venous thrombosis group, and the incidence of deep venous thrombosis was 8.2%. There were significant differences in age, fracture site, surgery modality, occupation type, operation time, smoking status, hospital stay and postoperative exercises between the two groups (p<0.05), but no significant differences in gender, drinking status, coronary heart disease, diabetes and hypertension (p>0.05). In multivariate analysis, old age greater than 50 years, arthroplasty and operation time more than three hours were independent risk factors, while physical labor and postoperative exercises were protective factors for deep venous thrombosis in lower extremity fractures.

**Conclusion:**

Fracture site was correlated to the incidence of deep venous thrombosis, old age, longer operation time, and arthroplasty were independent risk factors, physical labor and postoperative exercises were protective factors for deep venous thrombosis in patients with lower extremity fractures.

## INTRODUCTION

Deep venous thrombosis (DVT), as a common complication in lower extremity fractures^1^, can cause high mortality once pulmonary embolism occurs.^2^ It is estimated that 200 000 people die from pulmonary embolism each year.^3^ In clinical practice, DVT is often asymptomatic and in many patients the fatal pulmonary embolism is usually the first clinical manifestation.^4^ Many prophylactic methods for DVT, such as low dose heparin, aspirin, elastic stockings and intermittent pneumatic calf compression, have been employed widely in orthopedics department, but DVT is still a common dangerous postoperative complication.^5^ Subsequently, it is critical to study its distribution characteristics and related risk factors of DVT in lower extremity fractures to prevent and reduce its occurrence in orthopedics department.

In previously published literature, the distribution of DVT in lower extremity fractures was studied. Some authors concluded that hip fractures presented with a highest incidence of 17-58%^6^, the incidence in distal femur fractures treated surgically was 25%^7^, and in foot and ankle surgery was 2.1%.^3^ It seems that DVT may occur more often in the proximal fracture of lower extremity, and we speculate that fracture site may be correlated closely with the incidence of DVT in lower extremities. However, the abovementioned studies which came from different countries were carried out by different medical centers. Many factors such as prophylactic chemoprophylaxis, postoperative exercises and other measures may influence the occurrence of DVT. Subsequently, it is difficult to draw a definite conclusion. In a study of 102 patients with lower-extremity fractures, Abelseth found 40% DVT were of the femoral shaft, 43% of the tibial plateau, 22% of the tibial shaft, and 12.5% of the tibial plafond, suggesting a higher DVT incidence in more proximal fractures.^8^ Nevertheless, in Abelseth’s study the sample size was small, and some patients had bilateral lower-extremity fractures, which may affect the clinical conclusion adversely.

Moreover, some authors studied the risk factors related to DVT in lower extremity fractures. In a study of one hundred fifty-nine patients, Williams suggested that older age was a risk factor for DVT, but body mass index and sex were not significant predictors.^9^ In another study of nine hundred one consecutive patients, Park^10^ suggested an advanced age, cardiovascular disease, and chronic lung disease were independent risk factors.Michetti^11^ and Chen^12^ also analyzed the risk factors of DVT in fracture patients. However, there were controversial conclusions in studies, and it is necessary to perform more studies to clarify these issues. In addition, we believe that a further analysis of risk factors for DVT may facilitate to explain the possible correlation between fracture site and incidence of DVT in lower extremity fractures.

Therefore, in the present study, we retrospectively reviewed the patients treated surgically in orthopedics department of our hospital from May 2012 to July 2017, and our objectives were: (1) to clarify if fracture sites were correlated closely to DVT, and (2) to determine the risk factors of DVT in lower extremity fractures, to help surgeons make systematic prophylaxis of the fatal disease correctly.

## METHODS

In the current study, nine hundred and eighty-seven patients with lower extremity fractures treated surgically in the orthopedics department of our hospital from May 2012 to July 2017 were reviewed retrospectively. The clinical data including age, gender, fracture site, surgery modality, hospital stay, operation time, occupation type, hypertension, coronary heart disease, diabetes, smoking status, drinking status, postoperative exercises were collected.

The inclusion criteria of the study were:

1. Patients who were diagnosed with fracture of lower extremity and treated surgically
2. Patients with single fracture
3. DVT was confirmed by auxiliary examinations including Doppler ultrasound, magnetic resonance imaging venography and CT angiography.

The exclusion criteria of the study were:

1. Patients with multiple fractures.
2. Patients who required chronic dialysis, therapeutic anticoagulation for any reason, or patients with malignant tumor.
3. Patients who had DVT diagnosed before fracture, or who developed arterial thrombosis.

### Statistical analysis

Statistical analysis was conducted with SPSS 21.0 (SPSS Inc., Chicago, IL, USA). The comparison of measurement data was carried out by analysis of variance and enumeration data by Chi square test between groups. Multivariate logistic regression analysis was employed to find the correlation between variables and DVT. P < 0.05 was regarded as statistical significance.

## RESULTS

Nine hundred and eighty-seven patients with lower extremity fractures were treated surgically in the department of orthopedics. Among these cases, one hundred and fifty-eight patients were excluded for failing to meet the inclusion criteria, and 829 patients were included for analysis. In the 829 cases, 68 were diagnosed as DVT and included in DVT group, the other 761 were ruled out of the diagnosis of DVT and included in the Non-DVT group. The incidence of DVT was 8.2% in the current study.

**Fig.1:**
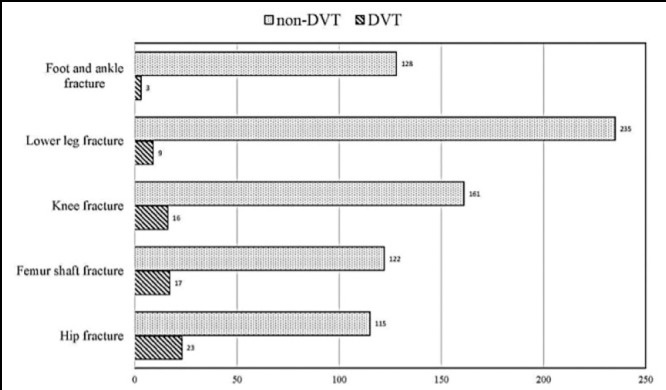
The distribution of DVT in different fracture sites.

The baseline characteristics of the patients in DVT and Non-DVT groups are shown in Table-I. In 829 patients, 138 were hip fracture, 139 were femur shaft fracture, 177 were knee fracture, 244 were lower leg fracture and 131 were foot or ankle fracture, and the DVT cases were 23, 17, 16,9 and 3, the rate of DVT were 16.7%, 12.2%, 9%, 3.7% and 2.3%, respectively. There were significant differences in rate of DVT among patients with different fracture sites (p<0.05), and hip fracture group presented with the highest rate of DVT (Fig.1).

**Table-I:** The baseline characteristics of the patients in DVT and Non-DVT groups.

Factors	DVT	Non-DVT	P-value
	n=68	n=761	
Age (> 50 years) (n,%)	49(72.1%)	392(51.5%)	0.001
Gender (M/F)	48/20	498/263	0.39
Fracture site			0.000
Hip (n, %)	23(33.8%)	115(15.1%)	
Femur shaft(n,%)	17(25%)	122(16.0%)	
Knee (n,%)	16(23.5%)	161(21.2%)	
Lower leg (n,%)	9(13.2%)	235(30.8%)	
Foot or ankle (n,%)	3(4.4%)	128(16.8%)	
Surgery modality			0.0005
Internal fixation	48(70.6%)	657(86.3%)	
Arthroplasty	20(29.4%)	104(13.7%)	
Hypertension (n, %)	17(25%)	159(20.9%)	0.43
Coronary heart disease (n, %)	15(22%)	183(24%)	0.71
Diabetes (n, %)	11(16.2%)	98(12.9%)	0.44
Hospital stay(days)	20.8±10.4	12.5±8.7	0.02
Postoperative exercises (n, %)	13(19.1%)	308(40.4%)	0.0005
Operation time>3 hours (n, %)	32(47.1%)	228(29.9%)	0.003
Smoking (n, %)	39(57.4%)	287(37.7%)	0.001
Drinking status (n, %)	28(41.2%)	262(34.4%)	0.26
Occupation type (Mental/Physical)	15/53	301/460	0.004

There were significant differences in age, fracture site, surgery modality, occupation type, operation time, smoking status, hospital stay and postoperative exercises between the two groups (p<0.05), but no significant differences in gender, drinking status, coronary heart disease, diabetes and hypertension (p>0.05, Table-I). Old age>50 years, hip fracture, arthroplasty, operation>3 hours, smoking status and longer hospital stay were risk factors (p<0.05), and physical labor and postoperative exercises were protective factors for DVT (p<0.05) in patients with lower extremity fractures (Table-II).

**Table-II:** The risk factors and protective factors for deep venous thrombosis.

Factors	P-value	OR (95% CI)
Age (> 50 years)	0.001	2.428(1.403-4.201)
Hip fracture	0.000	2.889 (1.683-4.958)
Operation time>3 hours	0.003	2.078(1.259-3.429)
Physical labor	0.03	0.433(0.239-0.781)
Arthroplasty	0.01	2.632(1.502-4.613)
Smoking	0.001	2.221 (1.344-3.671)
Postoperative exercises	0.000	0.348(0.187-0.647)

In multivariate analysis, old age greater than 50 years, arthroplasty and operation time more than three hours were independent risk factors, while physical labor and postoperative exercises were protective factor for DVT in patients with lower extremity fractures.

## DISCUSSION

In this study, we performed a retrospective analysis of 829 patients with lower extremity fracture to determine the correlation between fracture site and DVT as well as the risk factors for DVT. This study may help orthopedic surgeons make treatment strategy and prevent the occurrence of this disease correctly.

In the study, the incidence of DVT was 8.2%, which is lower than that in Abelseth’s study.^8^ However, we found the rate of DVT was 16.7%, 12.2%, 9%, 3.7% and 2.3% in hip, femur shaft, knee, lower leg and foot and ankle, respectively. This suggested a higher DVT incidence in more proximal fractures, indicating a similar result as Abelseth.^8^ In addition, we found in 829 patients hip fracture presented with the highest rate of DVT, and there were significant differences in the rate among patients with different fracture sites, demonstrating that hip fracture was a risk factor for DVT.

On the other hand, we found old age more than 50 years, arthroplasty and operation time more than three hours were independent risk factors for DVT, but hip fracture was not. In our opinion, most hip fractures, such as femoral neck fracture and trochanteric fracture, usually occur in old patients and many of them were treated using hip arthroplasty. Subsequently, we believe that it is old age and arthroplasty which lead to a higher incidence of DVT in patients with hip fracture. Many authors also advocated that old age was an important risk factor for DVT because of vascular sclerosis, high blood viscosity and poor venous valve function in old patients.^4^,^13^,^14^ At the same time, in a retrospective study of 276 old patients with hip fracture, Li also suggested that hip arthroplasty and longer operation time had significant correlation with the occurrence of DVT, highlighting the similar viewpoints.^13^

Moreover, we found that physical labor and postoperative exercises were protective factors for DVT. In our previous study of 376 postoperative neurosurgical patients, we had the same conclusion.^4^ Those mental workers usually keep on sitting in their daily life, and the prolonged work- and computer-related seated immobility was significantly associated with an increased risk of DVT. In terms of the postoperative exercises, in a study of 80 patients, Tominaga found that the preoperative walking disability was risk factor for DVT and VTE, and gait training during the early postoperative period is recommended to prevent VTE, also suggesting the same viewpoints.^15^

However, there were different viewpoints. In a study of 454 patients over 60 years old with surgically treated hip fractures, Wong found there was no significant correlation between DVT and age, type of fracture and operation.^16^ Additionally, Lee in his study found female gender was another risk factor for DVT in patients with lower extremity fractures^14^, and Guo concluded in his study of 196 patients the risk factors for DVT included the presence of a tumor, an age greater than 50 years, hypertension, and immobility.^17^ However, in our study we found no significant differences in the distribution of gender and hypertension between DVT and non-DVT group. In our opinion, many factors may affect the results of these studies, and the major reason may be attributed to the different inclusion and exclusion criteria.

### Limitations of the study

DVT of lower extremity was confirmed by different auxiliary examinations, including Doppler ultrasound, magnetic resonance imaging venography, or CT angiography. As the sensitivity of these auxiliary examinations are different^18^, which may affect the incidence of DVT, but our study didn’t include the detailed information about the diagnostic methods. Second, we concluded that old age, operation time and arthroplasty were independent risk factors of DVT, but some other factors may also affect the occurrence of DVT in patients with lower extremity fractures, which were not referred to in this study. Despite these limitations, we believe that fracture site was significantly correlated to the incidence of DVT, but old age, longer operation time, arthroplasty were independent risk factors, physical labor and postoperative exercises were protective factors for DVT in patients with lower extremity fractures.

### Ethical Committee

The study was approved by the ethics committee of our hospital.

### Authors’ Contribution

**QL, LL** conceived, designed and did statistical analysis & editing of manuscript.

**QL, XC, YYW and LL** did data collection and manuscript writing, and performed review and final approval of manuscript.
